# Advanced Characterization of Ceramic State Polymer Electrolyte at Radio Frequencies

**DOI:** 10.3390/polym14163345

**Published:** 2022-08-17

**Authors:** Wei Quan, Mohammed Nurul Afsar

**Affiliations:** Department of Electrical and Computer Engineering, Tufts University, Medford, MA 02155, USA

**Keywords:** non-contact measurement, dielectric, lithium, ionic relaxation, polymer electrolyte

## Abstract

Two newly developed non-contact dielectric measurement techniques were applied to characterize the complex permittivity spectra of a ceramic state polymer electrolyte. The Capacitance Bridge was employed to measure the electrolyte sample in a frequency range from 50 Hz to 20 KHz with a novel air gap method. The newly designed in-waveguide measurement by VNA (vector network analyzer) was applied to measure the electrolyte in the frequency range from 8.2 GHz to 40 GHz. Both methods are newly developed non-contact techniques and there was no physical contact on the polymer sample film surface during the measurement. The weak contact error in traditional measurement could be completely avoided in these non-contact methods. The ionic conductivity and complex electrical conductivity can be derived from the accurate complex dielectric spectra.

## 1. Introduction

Current energy cell design needs to take many factors such as safety and energy density into consideration. The electrolyte acts as one of the most important parts in the energy storage device. As a medium, the electrolyte is placed between anode and cathode to help transfer ions in every working cycle of the battery. This novel polymer electrolyte required both excellent ion conduction and current separation in the same medium structure design, which normally was a thin membrane with hundreds of micrometer thickness [1]. As a polymer electrolyte, this novel ceramic state electrolyte also performs a significant advantage over liquid electrolytes in terms of safety, weight, flexibility, and process ability.

**Figure 1:**
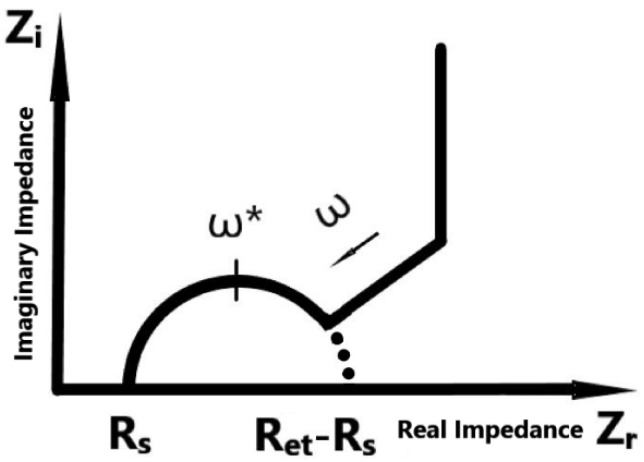
An ideal Nyquist curve of dielectric materials. The frequency ω increases following the arrow direction. ω* indicates the frequency at highest point of the semicircle.

**Figure 2:**
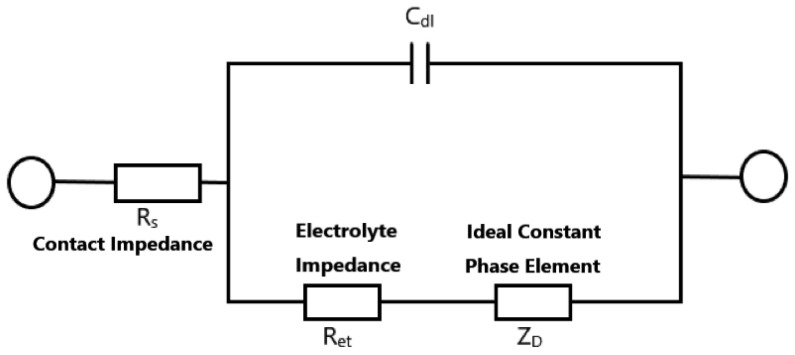
The equivalent circuit model of the ideal Nyquist curve.

Normally, the characterization of electronic and ionic conductivity is one of the most required steps in polymer electrolyte application [2]. In the traditional process, this measurement can not avoid contacting the electrolyte sample with testing electrodes by using AC impedance spectroscopy [3,4,5]. The measured complex impedance can be traced in the Nyquist plot shown in Figure 1. The semicircle in the complex impedance plane could represent the ideal impedance of the electrolyte. With the characteristics of the Nyquist plot, the ionic conductivity can be derived by using an equivalent circuit model shown in Figure 2.

The traditional contact measurement methods can not avoid contact impedance and error from partial contact problems because of physical contact between electrolyte and electrodes [6,7]. A series of newly developed non-contact dielectric measurements were firstly improved and applied in this novel ceramic state polymer electrolyte. The conductivity was successfully derived over a frequency range from 50 Hz to 20 KHz and from 8.2 GHz to 40 GHz.

The measurement in the frequency range 50 Hz to 20 KHz was using a capacitance bridge Andeen-Hagerling 2700A Bridge with an LD-3 dielectric capacitance cell. The measurements in the frequency range 8.2 GHz to 40 GHz were based on an Agilent 8510C vector network analyzer with eight different newly designed waveguides. The complex impedance spectra and complex conductivity spectra were derived from complex permittivity data obtained by these two measurements. With the same test process, the ionic relaxations can also be obtained by analyzing the loss tangent spectra.

## 2. Materials and Methods

**Figure 3:**
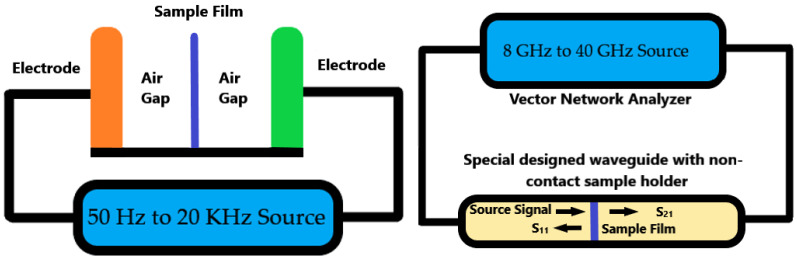
The Simplified diagram for non-contact measurement: air-gap capacitor technique for 50 Hz~20 KHz (**left**); in-waveguide technique for 8 GHz to 40 GHz (**right**).

A 12 mil (300 um) thickness electrolyte sample was prepared before the test. The whole series of experiments was processed in two frequency ranges, 50 Hz to 20 KHz and 8.2 GHz to 40 GHz. The measurement in these two frequency ranges used an air-gap capacitor method and in-waveguide method separately. Both methods are newly developed non-contact techniques. There was no physical contact on the electrolyte sample film surface during both measurements. The simplified diagram of both the measurement is shown below in Figure 3.

Most electronic properties of electrolytes could be evaluated from their dielectric permittivity spectra. Normally the dielectric permittivity can be written as $\hat{\varepsilon }(\omega )=\varepsilon^{\prime }(\omega )-j\varepsilon^{\prime \prime }(\omega )$. Where *ε*′ is the real part of permittivity, which is also called the dielectric constant, *ε*′ is the imaginary part and is called dielectric loss.

The current density in electrolyte with an external electric field can be presented as:

(1) $$J_{total}=J_{conduction}+J_{displacement}=\hat{\sigma }E=-i\omega \hat{\varepsilon }E$$

where *J_conduction_* is the conduction current, *J_displacement_* is the displacement current, and is the complex conductivity. After transformation, we can calculate the complex conductivity as:

(2) $$\hat{\sigma }=\sigma_{dc}+\sigma^{\prime }+j\sigma^{\prime \prime }=\sigma_{dc}+\omega^{n}\varepsilon^{\prime \prime }+j\omega \varepsilon^{\prime }$$

In this equation, *σ_dc_* presents the electronic conductivity of the electrolyte at a direct current. For a solid polymer material, this part of conductivity is extremely small. The activation energy applied in the test would determine the coefficient *n*. Therefore, the dominant part in the complex conductivity formula is the imaginary part of permittivity. It is also the real part of complex AC conductivity σ’ in the same equation. This part is frequency dependent and has a DC ionic conductivity plateau in a frequency range. This value can be evaluated at the loss tangent peak to find the ionic conductivity of the polymer electrolyte.

Instead of directly measuring conductivity or impedance as in the traditional technique, capacitance, in this measurement, distance, transmittance, and reflectance were the direct measurement results. After that, the permittivity, loss tangent, and conductivity can be derived with these measured results.

In the two certain electromagnetic field environments in this paper, in the air gap inside the capacitor and space inside the electromagnetic waveguide, the electrolyte sample film was inserted and the input/output microwave signal was detected and analyzed to calculate the dielectric permittivity of the sample. The real part and imaginary part of complex permittivity can be used to deduce the conductivity of the sample then.

A newly designed sample holder and microwave waveguide were employed with an air-gap capacitor and vector network analyzer. The whole measurement was based on the electromagnetic field and microwave signal analysis. There was no physical contact between signal source electrodes and sample film surface during the test. The result measured by these non-contact methods is much more accurate because the contact impedance and weak contact error could be totally avoided. Furthermore, there are no chemical reactions between materials of electrolytes and electrodes.

A Air Gap Capacitor

**Figure 4:**
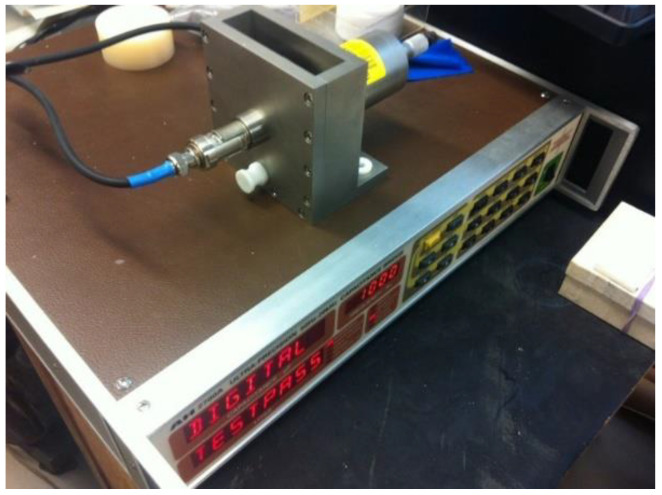
The air-gap capacitance bridge equipment setup.

For the measurement of the real part of permittivity (*ε*′) and the imaginary part of permittivity (*ε*″), an experimental setup is shown in Figure 4. As an energy storage component, the *ε*′ of electrolyte stands for its storage capability and *ε*″ represent the loss of energy. Moreover, the loss tangent or dissipation factor was used to determine the ratio, tan *δ* = *ε*″/*ε*′.

Compared to other chemical or physical methodology, this dielectric characterization can be much more easily processed. The convenience and accuracy make them a great measurement method for this kind of solid material. As we know, the real part of the dielectric constant can be calculated by *ε*′ = *C_x_*/*C*_0_, where *C*_0_ is capacitance in a vacuum and *C_x_* is capacitance with the inserted electrolyte. With this definition, we can easily conclude that electrolytes require high dielectric permittivity for better energy storage.

**Figure 5:**
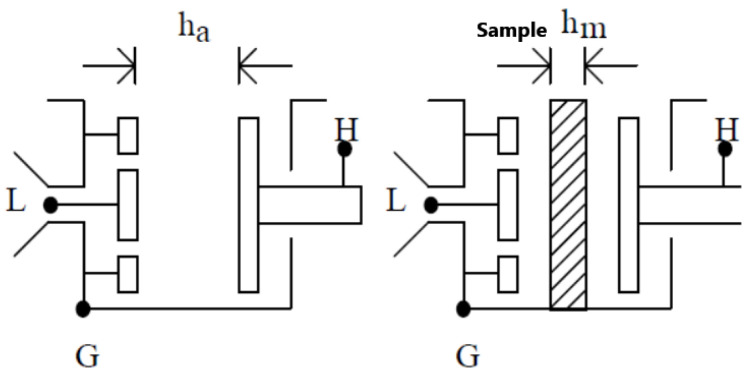
Diagram of the air-gap capacitance bridge technique. G stands for ground. L and H indicate the two electrodes.

In practical measurements, the electrodes are normally pressed by clamping on the electrolyte sample to avoid a weak contact problem, however, the contact problem can not be totally avoided. In our non-contact measurement process, we prepared the specimen with a designed thickness for further accurate characterization. The average thickness of sample *h_m_* would be measured by placing the specimen in some different positions, as shown in Figure 5. During the test, the space between electrodes would be set to 100 to 200 um longer than the thickness of the sample, as *ha*. Two groups of the capacitor and dissipation factor were measured with the sample inserted and without, *C_xa_*/tanδ*_xa_* and *C_a_*/tanδ*_a_*. The dielectric constant can be derived by:

(3) $$\varepsilon \prime =\frac{1}{1-\frac{C_{xa}-C_{a}}{C_{xa}}\frac{h_{a}}{h_{m}}}$$

The dissipation factor tanδ is calculated from

(4) $$\tan\delta =\tan\delta_{xa}+\left(\frac{h_{a}}{h_{m}}-1\right)\varepsilon^{\prime }\left(\tan\delta_{xa}-\tan\delta_{a}\right)$$

In this measurement, normal error in the contact method can be totally avoided. The accuracy in the preparation of specimen thickness *h_m_*, and measurement of holder space length *h_a_* becomes the main accuracy limitation factor. Repeating the measurement process with the sample placed in different position could significantly improve the accuracy.

B In-Waveguide Method

**Figure 6:**
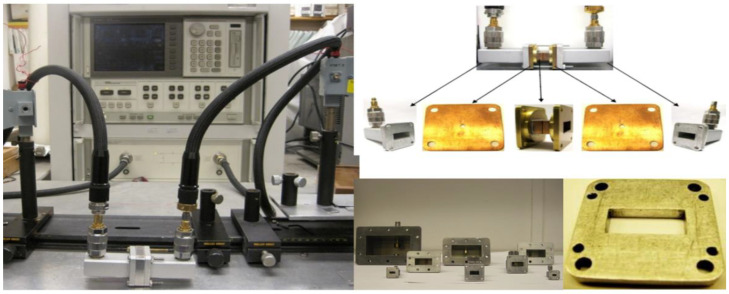
The in-waveguide setup using the newly designed eight waveguides and non-contact sample holder.

**Figure 7:**
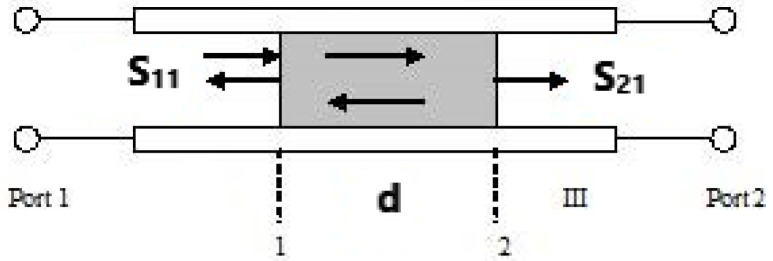
Diagram of in-waveguide experimental setup.

For the measurement in frequency range from 8.2 GHz to 40 GHz, the in-waveguide measurement was setup as shown in Figure 6. The diagram of how specimen placed was shown in Figure 7. S-parameter was measured for the inserted sample [8,9].

Before the practical measurement, the reference planes need to be set by using TRL (thru-reflect-line) calibration program with the typical quarter wavelength difference (l). The S-parameters in this measurement can be calculated as:

(5) $$\begin{array}{l} {\tilde{S}}_{11}=S_{11}e^{j(0\times \sqrt{k_{0}^{2}-k_{c}^{2}})}\\ {\tilde{S}}_{21}=S_{21}e^{j(l-d)\times \sqrt{k_{0}^{2}-k_{c}^{2}}} \end{array}$$

In this equation, *d* is the thickness of the specimen to be tested, *k_c_* is the cutoff wavenumber, *k*_0_ is the sample’s wavenumber, and *l* refers to the quarter wavelength difference between thru and line. The effect of *d* is much smaller than the holder’s thickness was taken into consideration in this calculation.

This measurement setup can negate any undesired effect from the waveguide itself, because these calibration process can help achieve less than –50 dB return losses inside the waveguide. With the direct measurement results, the reflection and transmission can be derived as:

$$\Gamma =K\pm \sqrt{K^{2}-1}$$

(6) $$K\;=\frac{{\tilde{S}}_{11}^{2}-{\tilde{S}}_{21}^{2}+1}{2{\tilde{S}}_{11}}$$

$$K\;=\frac{{\tilde{S}}_{11}^{2}-{\tilde{S}}_{21}^{2}+1}{2{\tilde{S}}_{11}}$$

The transmission coefficient through the material may also be written as *T* = e^−*γd*^ = e^−(α + *j*β)*d*^. The propagation constant through the material inside the waveguides has been derived to be:

(7) $$\gamma_{TE_{10}}=\frac{\ln\left(\frac{1}{\left|T\right|}\right)}{d}+j\left(\frac{2\pi n-\varphi_{T}}{d}\right)$$

After analyzing the above equations and boundary conditions, the propagation constant can present as:

$$\gamma_{TE_{10}}=j2\pi \sqrt{{\left(\frac{1}{\lambda_{0}}\right)}^{2}-{\left(\frac{1}{2a}\right)}^{2}}\cdot \sqrt{\mu \varepsilon }=j\Upsilon_{TE_{10}}^{0}\frac{\mu }{\eta }$$

(8) $$\gamma_{{TE}_{10}}^{0}=\sqrt{{\left(\frac{1}{\lambda_{0}}\right)}^{2}-{\left(\frac{1}{2a}\right)}^{2}}$$

The complex dielectric permittivity can be derived by utilizing these equations [10]. The final permittivity in this measurement is shown as:

(9) $$\begin{array}{l} \varepsilon =-j{\left(\frac{c}{f}\right)}^{2}\left(\frac{1-\Gamma }{1+\Gamma }\right)\left(\frac{1}{2\pi d}\right)\left(\ln(\frac{1}{\left|T\right|})+j(2\pi n-\phi_{T})\right)\\ \cdot \left(\sqrt{{\left(\frac{1}{\lambda_{0}}\right)}^{2}-{\left(\frac{1}{2a}\right)}^{2}}\right) \end{array}$$

## 3. Results

**Figure 8:**
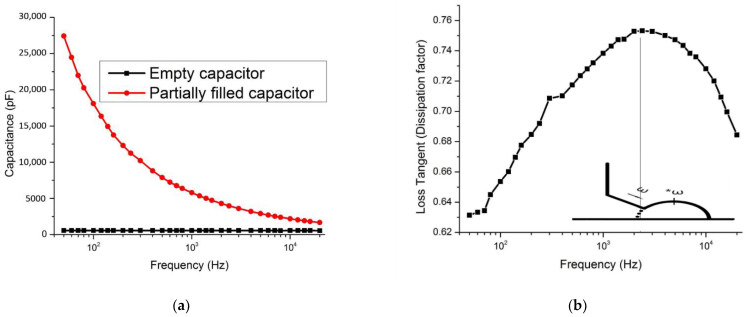
(**a**). The capacitance of the empty capacitor and capacitor with the inserted sample. (**b**). A peak of loss tangent indicates the ionic relaxation. An ideal Nyquist curve was inserted to make a comparison. The frequency ω increases following the arrow direction. ω* indicates the frequency at highest point of the semicircle.

**Figure 9:**
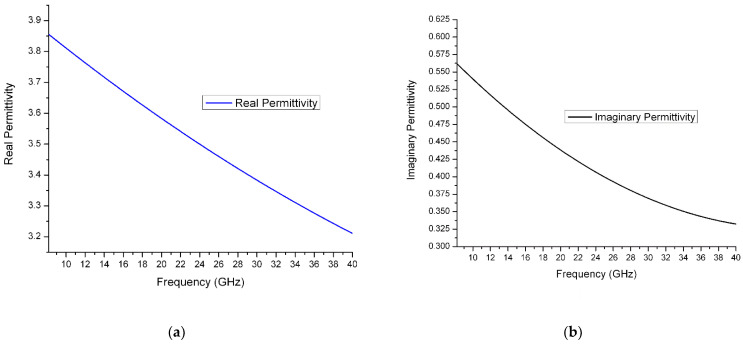
(**a**) Real part of relative permittivity of solid polymer electrolyte from 8.2 GHz to 40 GHz. (**b**) Imaginary part of relative permittivity of solid polymer electrolyte from 8.2 GHz to 40 GHz.

**Figure 10:**
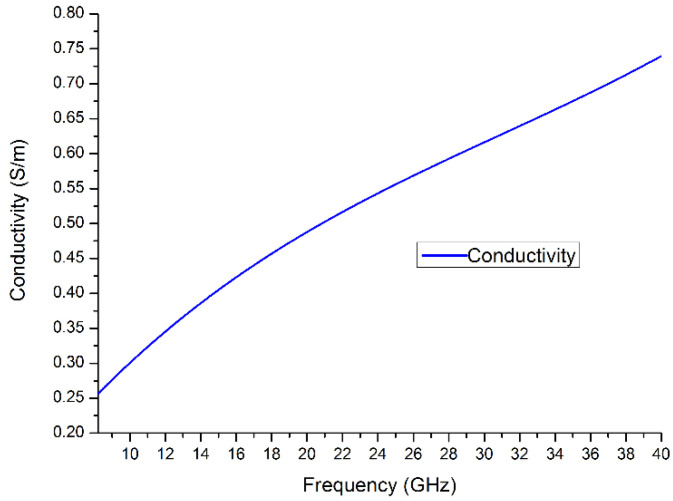
Conductivity spectra of solid polymer electrolyte from 8.2 GHz to 40 GHz.

In measurements using the air-gap capacitance bridge technique, the capacitance and loss tangent spectra were derived and plotted in Figure 8a,b. The data was collected and calculated in frequency from 50 Hz to 20 kHz. In measurement using the new designed in-waveguide technique, the reflection and transmittance can be measured. After calculation using collected data, the complex permittivity and conductivity can be derived and is shown in Figure 9a,b and Figure 10, separately. The final plot is close to a linear function with frequency increasing.

**Figure 11:**
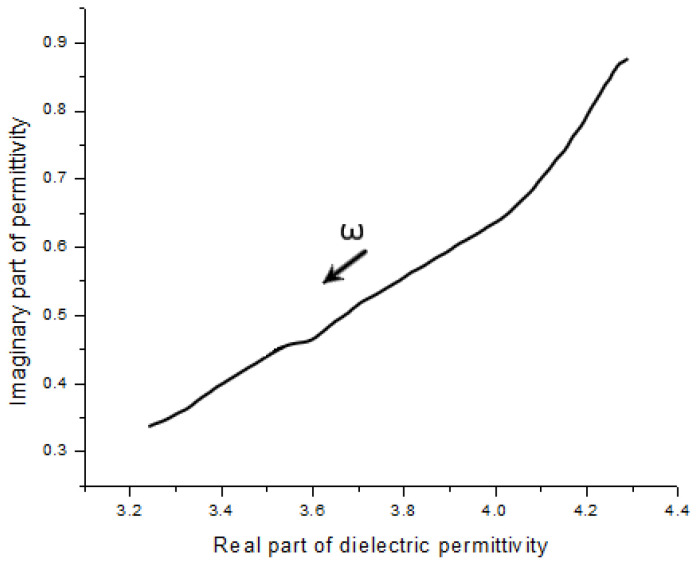
Cole–Cole curve of the polymer specimen.

In this ceramic state polymer electrolyte, the ions would normally move in two ways, as local vibrations and diffusion [11]. Because the external electric field changes rapidly in the high-frequency range like GHz, the ions in this field condition would not be motivated enough to transport. This performance makes the ions in the electrolyte become doing vibration more than diffusion [12]. The relationship between ionic vibration and diffusion is shown as the slope of the plot in Figure 10. The result of this series of measurements indicates this novel ceramic polymer electrolyte achieved high conductivity close to other liquid and gel electrolytes. The performance of this polymer electrolyte was better at low temperature because many kinds of liquid electrolytes have serious working temperature limitations. The Cole–Cole plot of this polymer electrolyte is shown in Figure 11.

At low frequency, from dc to 100 Hz, the polymer electrolyte is required to perform high electrode polarization as an ionic conductive material [13]. From the loss tangent plot in Figure 8b, the peak was present at a frequency of around 2 KHz. For comparison with traditional ionic conductivity, the Nyquist plot is used to show the peak frequency is consistent. In polymer electrolytes, lithium ionic relaxation employs the ions as a single-ion carrier [14]. Moreover, the loss tangent peak could help to analyze the ionic relaxation time and diffusion activation energy at a different temperature [15]. Because the ionic relaxation is dominated in this solid electrolyte at a frequency from 8.2 GHz to 40 GHz, the dielectric permittivity decreases as the frequency goes up in this range.

## 4. Discussion

**Figure 12:**
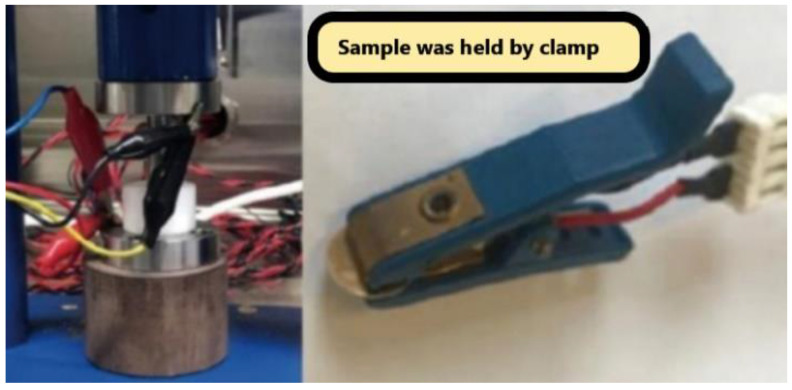
Setup for traditional ionic conductivity measurement.

For verification of this newly developed non-contact characterization technique, the same sample was measured by the traditional contact method, as in Figure 12, to make a comparison.

In the contact method, the sample film was held by a clamp with an AC source. The pressure on the clamp would obliviously affect the test result of the electrolyte because of weak contact errors. The conductivity measured by this method was about 30% lower than the non-contact method result. This is reasonable because the incomplete contact would result in more AC impedance. This weak contact impedance can be reduced by using better equipment with more stable pressure, however, this system error due to technique can not be fully avoided. The samples with different thickness, area, and roughness would result in a big difference in contacting force. The test result with this contact method has poor consistency and low reproducibility.

In contrast, the non-contact measurement could avoid the contact impedance and weak contact problem in traditional methods. However, there is still some uncertainty in both the air-gap capacitor and in-waveguide techniques. The random error of capacitance was 10^−3^ pF, and the random error of the dissipation factor was 10^−5^; both are much higher than what we expected from the instruments, which should be 10^−5^ pF and 1.5 × 10^−7^ for Andeen-Hagerling 2700A Bridge and loss tangent separately. In addition, the return loss in Agilent 8510C VNA was less than −50 dB which was worse than designed. This makes a 1% uncertainty belong to measured dielectric permittivity. These uncertainties mostly come from instrument accuracy limitations, which can be improved by upgrading measurement equipment in the future.

## 5. Conclusions

In this article, a new dielectric characterization methodology was presented on the materials which may have electrical applications, such as polymer electrolytes. Due to the fact that most current ionic conductivity measurement methods require electrode contact on the electrolyte, even though this measurement can give direct conductivity results, weak contact error always exists and can not be fully avoided. No matter what kinds of equipment are used, we can not provide a perfect pressure on contact during measurement. Moreover, the pressure during experiments and practical application can not be completely matched, so the conductivity measured can be very different from practical application. Therefore, we developed this non-contact measurement to propose a new characterization methodology for dielectric properties.

In this paper, a ceramic state polymer electrolyte has been successfully characterized with a series of this newly developed non-contact measurement techniques. The measurements were implemented in two frequency ranges, 50 Hz to 20 KHz and 8.2 GHz to 40 GHz. The conductivity and ionic relaxation can be deduced from measured data as capacitance, loss tangent spectra, and dielectric complex permittivity. This non-contact measurement could be a powerful tool in the electric property characterization of polymer materials to replace traditional contact methods. The successful experience in this project can also be expanded to other applications of polymer not limited to electrolytes; any kind of application that requires a dielectric performance for polymers could be potential suitable situation for this non-contact measurement technique.

## Data Availability

The data presented in this study are available on request from the corresponding author.
